# Syntheses of novel pyridine-based low-molecular-weight luminogens possessing aggregation-induced emission enhancement (AIEE) properties

**DOI:** 10.3762/bjoc.18.60

**Published:** 2022-05-24

**Authors:** Masayori Hagimori, Tatsusada Yoshida, Yasuhisa Nishimura, Yukiko Ogawa, Keitaro Tanaka

**Affiliations:** 1 Faculty of Pharmaceutical Sciences, Mukogawa Women’s University, 11-68 Koshien Kyubancho, Nishinomiya 663-8179, Japan; 2 Graduate School of Biomedical Sciences, Nagasaki University, 1-7-1 Sakamoto, Nagasaki 852-8501, Japan; 3 Faculty of Pharmaceutical Sciences, Nagasaki International University, 2825-7, Huis Ten Bosch, Sasebo 859-3298, Japan; 4 Graduate School of Engineering, Nagasaki University, 1-14, Bunkyo-machi, Nagasaki 852-8131, Japan

**Keywords:** acceptor–donor–acceptor, AIEE, low molecular weight, one-pot reaction, ((pyridin-2-yl)amino)maleimide, TD-DFT calculation

## Abstract

Novel pyridine-based fluorescing compounds, viz. pyrido[1,2-*a*]pyrrolo[3,4-*d*]pyrimidines **3a**,**b** and *N*-methyl-4-((pyridin-2-yl)amino)maleimides **4a**–**e**, were selectively prepared by a one-pot reaction between a functionalized maleimide and 2-aminopyridines with electron-donating or electron-withdrawing groups at position 5 and were investigated photophysically and computationally. The photophysical studies revealed that all the synthesized compounds exhibited fluorescence in organic solvents, while *N*-methyl-4-((pyridin-2-yl)amino)-substituted maleimide derivatives **4a**–**e**, which are based on an acceptor–donor–acceptor (A–D–A) system, exhibited aggregation-induced emission enhancement (AIEE) properties in aqueous media. Compounds **4a** and **4e**, bearing electron-withdrawing groups (Br and CF_3_, respectively) showed 7.0 and 15 times fluorescence enhancement. Time-dependent density functional theory (TD-DFT) calculations were performed to gain better insight into the electronic nature of the compounds with and without AIEE properties.

## Introduction

Fluorescent compounds have attracted considerable attention as functional materials because of their applications in areas such as information devices, displays, and clinical diagnosis [1–3]. Organic compounds with planar structures and large π systems exhibit strong fluorescence in dilute solutions [4–5]. However, these compounds usually form aggregate structures in high-concentration solutions, and their emission efficiency, chromogenic properties, and light sensitivity decrease rapidly [5–6]. In recent years, contrary to conventional fluorescent compounds, aggregation-induced emission enhancement (AIEE)-based compounds that exhibit strong fluorescence in aggregate structures have been reported [7–10]. Because the aggregated state of AIEE-based compounds is affected by the external environment, these compounds have found use in clinical applications as chemical sensors or fluorescent probes [7–10].

Pyridine is a nitrogen-containing heterocyclic compound found in many bioactive substances and medicines as one of the basic core skeletons [11–12]. In addition, pyridine is an essential skeleton for fluorescent compounds, and fluorescence can be enhanced by optimizing the internal charge transfer (ICT) state of pyridine by introducing electron-donating or electron-withdrawing groups [13–15]. Previously, we have reported various pyridine derivatives, including polysubstituted pyridines and fused pyridines, which exhibited strong fluorescence in organic solvents (ethanol and dichloromethane) [16–19], while their fluorescence in aqueous media was quenched due to aggregation-caused quenching (ACQ) [10]. Generally, the restriction of intermolecular π–π interactions in highly planar compounds plays a key role in aggregate structures exhibiting fluorescence [5–6,10]. In addition, intramolecular mechanisms, such as intramolecular rotation (RIR), intramolecular charge transfer, and twisted intramolecular charge transfer (TICT) are involved in AIEE [20–22]. Various AIEE-based luminogens have been developed based on these mechanisms; however, many of them are high-molecular-weight compounds (MW > 500) with bulky substituents, which limits their clinical applications such as cell imaging and cell sorting.

To develop low-molecular-weight AIEE-based luminogens, we have synthesized a series of fluorescent compounds by the reaction of nucleophilic maleimides with 2-aminopyridines. This resulted in the development of a novel method to obtain heterocyclic compounds, such as ring-fused pyridines (pyrido[1,2-*a*]pyrrolo[3,4-*d*]pyrimidines) and secondary aminopyridines (*N*-methyl-4-((pyridin-2-yl)amino)-substituted maleimides), by changing the substituents at position 5 of the 2-aminopyridine. Interestingly, among these pyridine derivatives, secondary aminopyridines based on the acceptor–donor–acceptor (A–D–A) system exhibit AIEE properties in aqueous media, which may be novel candidate molecules for AIEE. Herein, we report the synthesis, photophysical properties, and computational studies of pyrido[1,2-*a*]pyrrolo[3,4-*d*]pyrimidines and *N*-methyl-4-((pyridin-2-yl)amino)-substituted maleimides.

## Results and Discussion

**Scheme 1:**
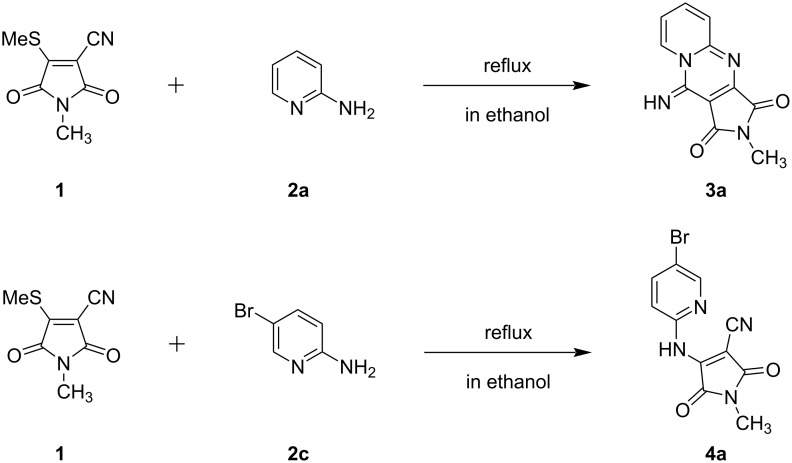
Syntheses of pyrido[1,2-*a*]pyrrolo[3,4-*d*]pyrimidine **3a** and *N*-methyl-4-((5-bromopyridin-2-yl)amino)-substituted maleimide **4a**.

**Figure 1:**
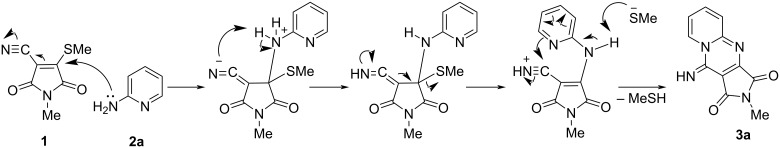
Presumed reaction mechanism to produce **3a**.

Maleimides are versatile reagents for the synthesis of heterocyclic compounds. Previously, we have reported functional maleimides derived from ketene dithioacetals and have prepared fluorescent compounds using this reagent [23–24]. In this study, we used 1-methyl-4-(methylsulfanyl)-2,5-dioxo-2,5-dihydro-1*H*-pyrrole-3-carbonitrile (**1**) with a methylsulfanyl group as a good leaving group. As shown in Scheme 1, the one-pot reaction of **1** with 2-aminopyridine (**2a**) proceeded by refluxing in ethanol for 2 h to produce the ring-fused pyridine compound, 10-imino-2-methylpyrido[1,2-*a*]pyrrolo[3,4-*d*]pyrimidine-1,3(2*H*,10*H*)-dione (**3a**), in 97% yield. The chemical structure of product **3a** was confirmed by ^1^H and ^13^C NMR spectroscopy (Figures S1 and S2 in Supporting Information File 1) and was obtained via the following reaction mechanism: nucleophilic attack of the amino group of 2-aminopyridine to maleimide **1**, followed by elimination of the methylsulfanyl group, and subsequent cyclization (Figure 1). The ring-fused pyridine compound **3b** was obtained from the reaction of **1** with 2-aminopyridine **2b**, which has an electron-donating methyl group at position 5 of the pyridine ring (Table 1). On the other hand, the reaction of **1** with 5-bromo-2-aminopyridine (**2c**) afforded an *N*-methyl-4-((pyridin-2-yl)amino)-substitued maleimide, 4-((5-bromopyridin-2-yl)amino)-1-methyl-2,5-dioxo-2,5-dihydro-1*H*-pyrrole-3-carbonitrile (**4a**), based on an A–D–A system containing two acceptor (maleimide and pyridine) and one donor (secondary amine) moieties in 72% yield (Scheme 1). Due to the electron-withdrawing effect of the bromo substituent at position 5 of the pyridine ring, the cyclization reaction did not occur. The structure of product **4a** was confirmed by ^1^H and ^13^C NMR spectroscopy (Figures S5 and S6 in Supporting Information File 1). Similarly, the reaction of **1** with 2-aminopyridine derivatives **2d**–**g** bearing electron-withdrawing groups, except for the nitro-group containing substrate **2h**, at position 5 of the pyridine ring, afforded *N*-methyl-4-((pyridin-2-yl)amino)-substituted maleimide derivatives **4b**–**e** (Table 1).

**Table 1:** Syntheses of pyrido[1,2-*a*]pyrrolo[3,4-*d*]pyrimidines **3a**,**b** and *N*-methyl-4-((pyridin-2-yl)amino)-substituted maleimide derivatives **4a**–**e**.

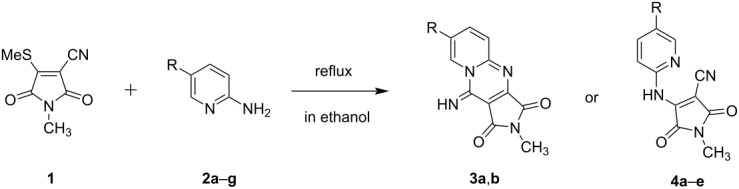				

entry	2-aminopyridine	R	yield (%)	product

1	**2a**	5-H	97	**3a**
2	**2b**	5-CH_3_	86	**3b**
3	**2c**	5-Br	72	**4a**
4	**2d**	5-F	68	**4b**
5	**2e**	5-CN	32	**4c**
6	**2f**	5-COOCH_3_	43	**4d**
7	**2g**	5-CF_3_	46	**4e**
8	**2h**	5-NO_2_	–	no reaction

Naka et al. previously reported the facile syntheses of *N*-alkyl-arylaminomaleimide derivatives as D–A system molecules by the reaction of dimethyl acetylenedicarboxylate with arylamines, which showed AIEE properties in 10% or 20% (v/v) THF aqueous solution [25]. In this reaction, the same aryl groups were easily introduced to the 4 and *N-*positions of maleimide. However, an additional step was required to synthesize *N*-alkyl-arylaminomaleimides bearing different aryl and alkyl groups. In addition, the synthesis of A–D–A-type molecules such as the introduction of pyridine rings has not been examined. Our simple one-pot method easily enables the introduction of a pyridine group to maleimide under mild conditions, affording the A–D–A-type molecules that are expected to be novel low-molecular-weight fluorescent materials in moderately good yields.

The UV–vis spectra of all compounds were recorded in ethanol (EtOH), a polar solvent, and in dichloromethane (DCM), a non-polar solvent (see Figures S15–S17 in Supporting Information File 1). The maximum absorption peaks (λ_max_) shifted slightly to longer wavelengths in dichloromethane. Table 2 summarizes the excitation maxima (Ex_max_), emission maxima (Em_max_), and fluorescence quantum yields (Φ) of the molecules from fluorescence spectroscopic studies. The pyrido[1,2-*a*]pyrrolo[3,4-*d*]pyrimidine derivatives **3a**,**b** possessing a highly complex ring-fused system emitted fluorescence at 545–546 nm and 537–538 nm in ethanol and dichloromethane (Figures S18 and S19 in Supporting Information File 1). The Φ value of **3b** increased with the introduction of a methyl group at position 5 of the pyridine ring of **3a** in both solvents, suggesting that the electron-donating effect of the methyl group stabilized the ring system and thus induced increased fluorescence. The *N*-methyl-4-((pyridin-2-yl)amino)-substituted maleimides **4a**–**e** comprised an A–D–A system, which exhibited an obvious substitution effect on the fluorescence properties (Figures S18 and S19 in Supporting Information File 1). Although the unsubstituted compound **4a** emitted weak fluorescence at 553 nm and 539 nm in ethanol and dichloromethane, respectively, the introduction of electron-withdrawing groups at position 5 of the pyridine ring of **4a** greatly affected the Ex_max_ value of products **4b**–**e** and induced hypsochromic shifts of about 72–82 nm in ethanol. In contrast, similar hypsochromic shifts were observed in dichloromethane only in case of compounds **4c** and **4e** bearing strong electron-withdrawing substituents (CN and CF_3_, respectively), and the Φ value of these compounds increased. These solvatochromic effects could be attributed to changes in the ICT state of the molecules, indicating that fluorescence properties of *N*-methyl-4-((pyridin-2-yl)amino)-substituted maleimides could be modulated by the electron push–pull effect of substituents.

**Table 2:** Fluorescence data for products **3a**,**b**, and **4a**–**e** in EtOH and DCM.

	dissolved in EtOH				dissolved in DCM		
			
compound	EX_max_ (nm)^a^	EM_max_ (nm)^b^	Φ^c^		EX_max_ (nm)^a^	EM_max_ (nm)^b^	Φ^c^

**3a**	468	545	0.01		471	537	0.03
**3b**	469	546	0.02		481	538	0.06
**4a**	476	553	0.01		477	539	0.01
**4b**	415	471	0.01		474	538	0.01
**4c**	377	481	0.01<		373	469	0.03
**4d**	378	481	0.01<		475	539	0.01<
**4e**	378	477	0.01		373	470	0.02

^a^Each excitation wavelength was determined by scanning at the fluorescence wavelength. ^b^Each emission was measured using excitation wavelengths. ^c^Fluorescence quantum yields were obtained by using an absolute PL quantum yield measurement system (C9920-1) of Hamamatsu Photonics.

**Figure 2:**
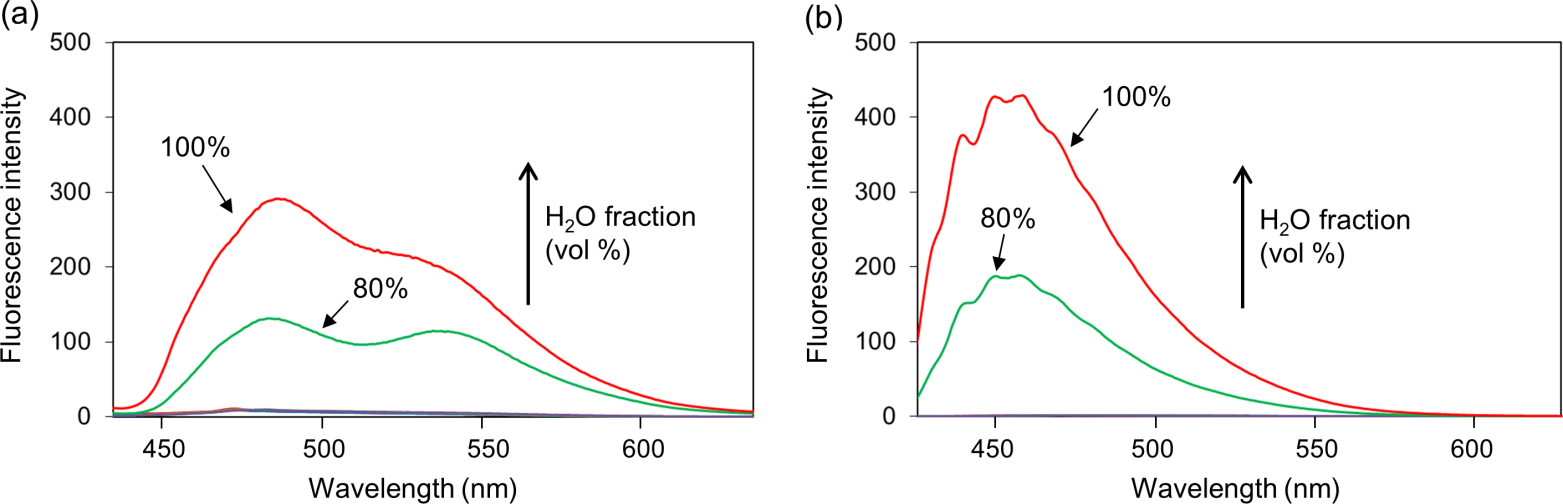
Fluorescence spectral profiles of (a) **4a** (10^−5^ M, Ex_max_ = 413 nm) and (b) **4e** (10^−5^ M, Ex_max_ = 415 nm) in H_2_O/EtOH mixture with different water fractions.

**Figure 3:**
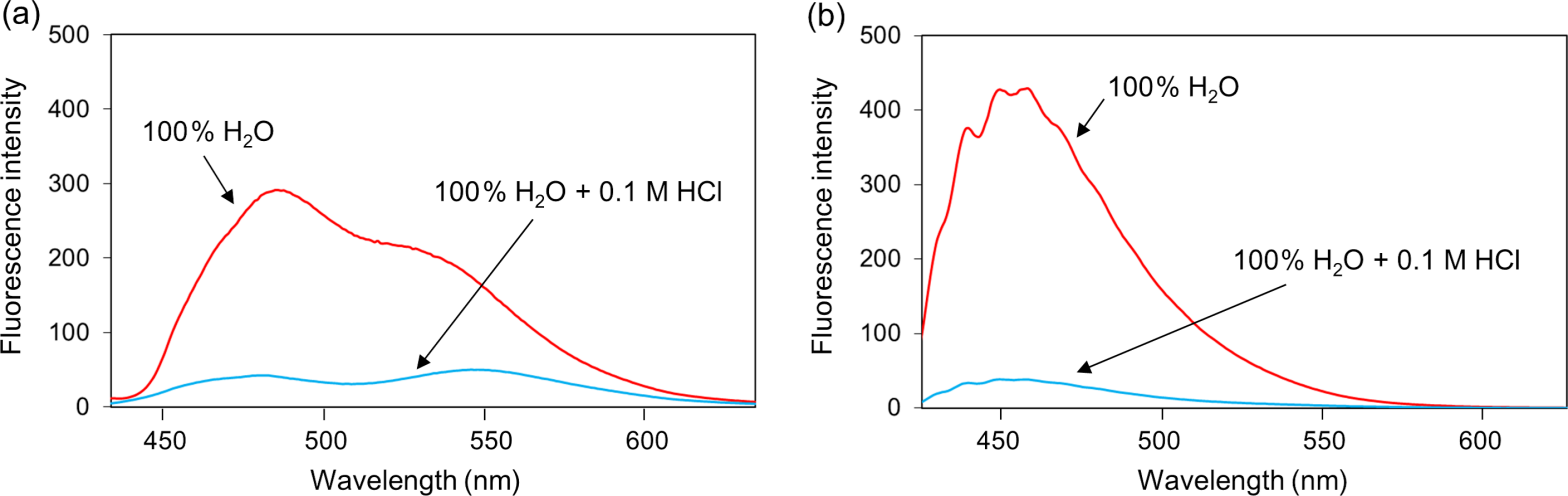
Fluorescence spectral changes of (a) **4a** (10^−5^ M, Ex_max_ = 413 nm) and (b) **4e** (10^−5^ M, Ex_max_ = 415 nm) upon addition of 0.1 M HCl.

**Figure 4:**
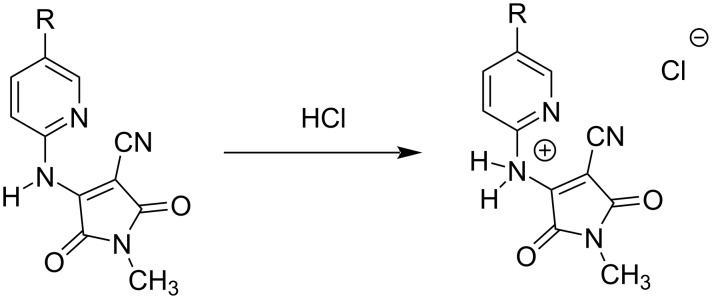
Protonation of *N*-methyl-4-((pyridin-2-yl)amino)-substituted maleimides **4** by 0.1 M HCl.

The AIEE properties of products **3a**,**b**, and **4a**–**e** were evaluated in different EtOH/H_2_O (v/v) solvent mixtures (Table 3 and Figure S20 in Supporting Information File 1). The UV–vis spectra of all compounds in H_2_O are shown in Figure S17 (Supporting Information File 1). The fluorescence intensities of the ring-fused compounds **3a**,**b** gradually decreased with increasing water fractions, and the ratio of water to ethanol Φ values (Φ_H2O_/Φ_EtOH_) was smaller than 1.0. These results indicated that aggregation was induced by π–π stacking interaction of the planar structures of compounds **3a** and **3b** in aqueous solution and that their excited states decayed by non-radiative pathways, resulting in ACQ. In contrast, the fluorescence intensities of compounds **4a**–**e** increased in 100% water, and the Φ_H2O_/Φ_EtOH_ value was higher than 1.0, indicating that AIEE occurred. In particular, the fluorescence of compounds **4a** and **4e**, bearing Br and CF_3_ groups, respectively, largely increased with the addition of H_2_O to an ethanolic solution (Figure 2) with Φ_H2O_/Φ_EtOH_ values of 0.07 and 0.12, respectively, which were 7.0 and 15 times higher than those in ethanol (non-aggregated form). Because compounds **4a**–**e** form an A–D–A system, charge disproportionation may affect intermolecular π–π interactions and lead to AIEE. The enhanced fluorescence was almost completely quenched by the addition of an 0.1 M HCl solution to solutions of compounds **4a** and **4e** (Figure 3). This result indicated that the protonation of the secondary amine disrupted the intermolecular π–π interactions or planarity of compounds 4 (Figure 4), and the rigid structure in solution was involved in AIEE.

**Table 3:** Fluorescence data for **3a**,**b**, and **4a**–**e** in H_2_O.

	dissolved in H_2_O			
		
compound	EX_max_ (nm)^a^	EM_max_ (nm)^b^	Φ^c^	Φ_H2O_/Φ_EtOH_^d^

**3a**	468	559	0.01<	1.0<
**3b**	355	550	0.01	0.5
**4a**	413	486	0.07	7.0
**4b**	431	553	0.02	2.0
**4c**	358	467	0.01	1.0>
**4d**	421	460	0.01	1.0>
**4e**	415	452	0.12	15

^a^Each excitation wavelength was determined by scanning at the fluorescence wavelength. ^b^Each emission was measured using excitation wavelengths. ^c^Fluorescence quantum yields were obtained by using an absolute PL quantum yield measurement system (C9920-1) of Hamamatsu Photonics. ^d^The ratio of Φ in H_2_O to Φ in EtOH.

**Figure 5:**
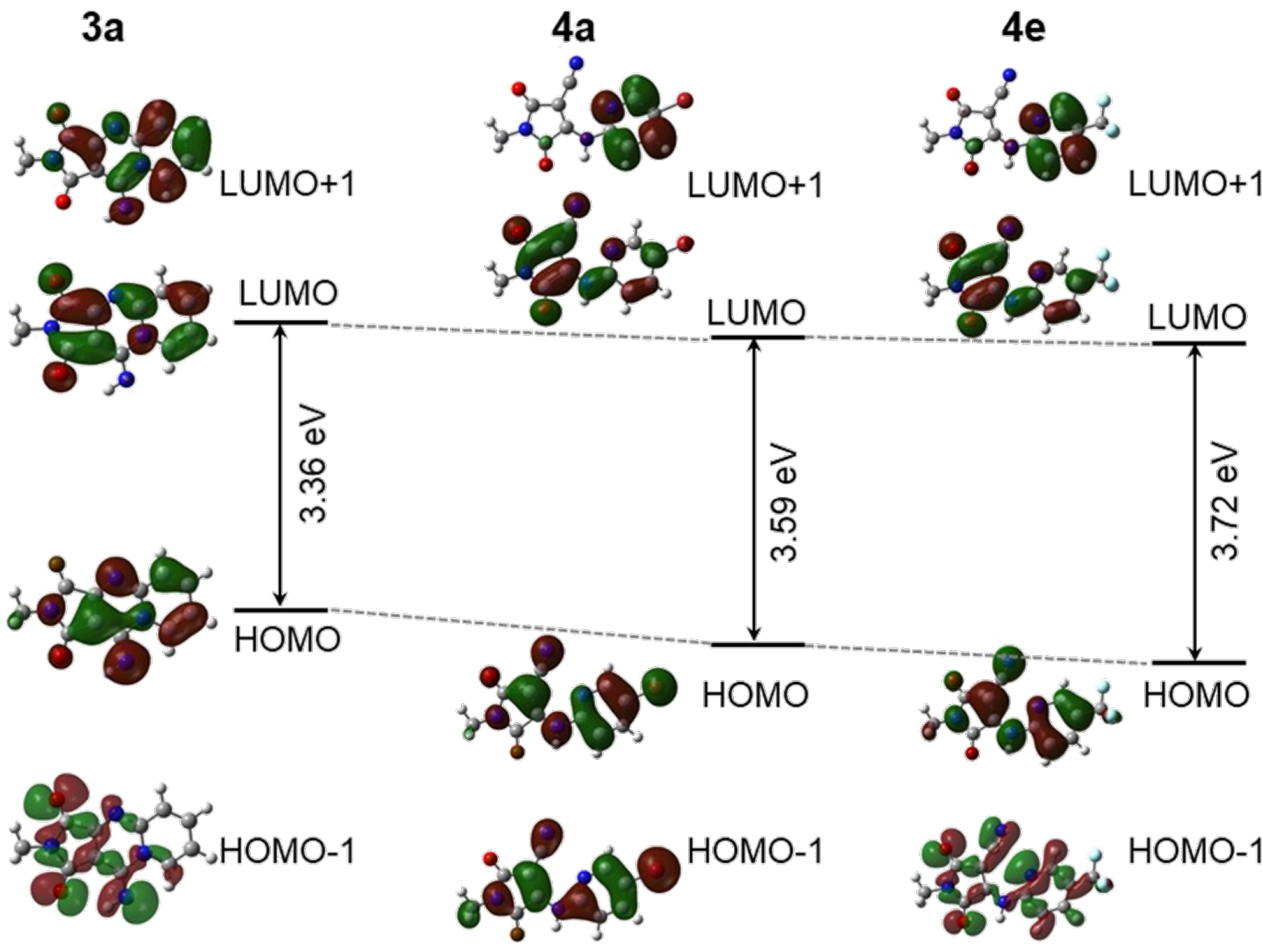
Frontier molecular orbitals and HOMO–LUMO energy gaps of compounds **3a**, **4a**, and **4e** for ground-state calculated by using the B3LYP/6-31G(d,p) level of theory in dichloromethane.

To compare the electronic natures of the compounds with and without AIEE, we performed TD-DFT calculations on each monomer of **3a**, **4a**, and **4e**. The graphical representations of the highest occupied molecular orbital (HOMO) and the lowest unoccupied molecular orbital (LUMO) for the ground-state geometries of each monomer are shown in Figure 5. The dihedral angles between the pyrimidine and maleimide rings, the amine linkage, were nearly zero degrees for **4a** and **4e** in all three solvents, adopting a highly planar structure as well as compound **3a**. The HOMO and LUMO were distributed on the entire structure of each compound owing to their high planarity in both the ground and excited states. The large overlap between these two frontier orbitals resulted in efficient absorption and emission.

The calculated vertical excitation energies of compounds **3a**, **4a**, and **4e**, along with their oscillator strengths, are listed in Table 4. The experimentally observed Ex_max_ values for the S_0_→S_1_ transition are somewhat different from the calculated ones, but the order of Ex_max_^calc^ corresponds to Ex_max_^obs^ other than in water, i.e. Ex_max_ (**4a**) > Ex_max_ (**3a**) > Ex_max_ (**4e**) in dichloromethane and ethanol. As anticipated, the S_0_→S_1_ transition in both compounds is mostly dominated by charge transfer from the HOMO to the LUMO, corresponding to a π→π* electron transition. There was no significant change (≤7 nm) in the Ex_max_ for compound **3a** in the three solvents: Ex_max_^obs^ ranged from 468 to 471 nm and Ex_max_^calc^ ranged from 464 to 468 nm. From calculations, a smaller solvent effect is seen in the Ex_max_^calc^ values for compound **4a** (484–488 nm) and **4e** (404–407 nm), whereas the Ex_max_^obs^ were found at 476 nm in ethanol and 413 nm in water for **4a** and at 378 nm in ethanol and 415 nm in water for compound **4e**, respectively. This discrepancy between the calculated and observed Ex_max_ values for compounds **4a** and **4e** suggests that aggregation occurs in water, and such behavior cannot be reproduced using the monomer model (non-aggregated form).

**Table 4:** Calculated excitation energies (*E* [Ex_max_]), oscillator strengths (*f*), and main components of the transition of the three lowest excited states for compounds **3a**, **4a**, and **4e** using the TD-B3LYP/6-311+G(d,p)//B3LYP/6-31G(d,p) level of theory.

solvent	compound	excited states	*E* (eV)	[Ex_max_^obs^]^a^ (nm)	[Ex_max_^calc^] (nm)	*f*	main components of the transition (% contribution)

DCM	**3a**	S_1_	2.67	[471]	[464]	0.224	HOMO→LUMO (98)
		S_2_	3.56	–	[348]	0.121	HOMO→LUMO+1 (97)
		S_3_	3.62	–	[343]	0	HOMO-1→LUMO (94)
	**4a**	S_1_	2.54	[477]	[488]	0.386	HOMO→LUMO (98)
		S_2_	3.83	–	[324]	0	HOMO-3→LUMO (62); HOMO-2→LUMO (36)
		S_3_	3.93	–	[316]	0.161	HOMO-1→LUMO (94)
	**4e**	S_1_	3.07	[373]	[404]	0.308	HOMO→LUMO (98)
		S_2_	3.79	–	[327]	0	HOMO-1→LUMO (50); HOMO-3→LUMO (47)
		S_3_	4.04	–	[307]	0.001	HOMO-3→LUMO (50); HOMO-1→LUMO (48)

EtOH	**3a**	S_1_	2.66	[468]	[466]	0.239	HOMO→LUMO (99)
		S_2_	3.57	–	[348]	0.140	HOMO→LUMO+1 (98)
		S_3_	3.64	–	[340]	0	HOMO-1→LUMO (94)
	**4a**	S_1_	2.55	[476]	[486]	0.409	HOMO→LUMO (98)
		S_2_	3.84	–	[323]	0	HOMO-3→LUMO (60); HOMO-2→LUMO (37)
		S_3_	3.92	–	[317]	0.167	HOMO-1→LUMO (94)
	**4e**	S_1_	3.05	[378]	[406]	0.329	HOMO→LUMO (98)
		S_2_	3.81	–	[326]	0	HOMO-1→LUMO (51); HOMO-3→LUMO (46)
		S_3_	4.05	–	[306]	0.001	HOMO-3→LUMO (51); HOMO-1→LUMO (47)

H_2_O	**3a**	S_1_	2.65	[468]	[468]	0.245	HOMO→LUMO (99)
		S_2_	3.57	–	[347]	0.148	HOMO→LUMO+1 (98)
		S_3_	3.65	–	[339]	0	HOMO-1→LUMO (94)
	**4a**	S_1_	3.10	[413]	[484]	0.420	HOMO→LUMO (98)
		S_2_	3.85	–	[322]	0	HOMO-3→LUMO (60); HOMO-2→LUMO (37)
		S_3_	3.92	–	[317]	0.169	HOMO-1→LUMO (94)
	**4e**	S_1_	3.04	[415]	[407]	0.339	HOMO→LUMO (98)
		S_2_	3.81	–	[325]	0	HOMO-1→LUMO (52); HOMO-3→LUMO (46)
		S_3_	4.05	–	[306]	0.001	HOMO-3→LUMO (51); HOMO-1→LUMO (47)

^a^Experimentally observed excitation wavelengths are listed in Table 2 and Table 3.

## Conclusion

To discover low-molecular-weight AIEE-based luminogens, we synthesized pyrido[1,2-*a*]pyrrolo[3,4-*d*]pyrimidines **3a**,**b** and *N*-methyl-4-((pyridin-2-yl)amino)-substituted maleimides **4a**–**e** in moderately good yields by reacting functionalized maleimides with 2-aminopyridines under mild conditions. The presence of electron-donating or electron-withdrawing groups at position 5 of the 2-aminopyridine greatly affected the fluorescence properties of the products, and *N*-methyl-4-((pyridin-2-yl)amino)-substituted maleimides, containing electron-withdrawing groups formed an A–D–A system and exhibited AIEE properties in aqueous media. In particular, compounds **4a** and **4e** bearing electron-withdrawing groups (Br and CF_3_, respectively) exhibited a large fluorescence enhancement. A comparison of the calculated and observed Ex_max_ using TD-DFT calculations revealed the basic features of compounds **4**. Therefore, we envision *N*-methyl-4-((pyridin-2-yl)amino)-substituted maleimides based on an A–D–A system as novel luminogens for AIEE and are currently investigating the underlying mechanism and future biological applications.

## Supporting Information

File 1 General information, synthesis of **3a**,**b**, and **4a**–**e**, experimental procedure of fluorescence, theoretical computation method measurements, NMR, UV–vis, and fluorescence spectra.
