# Supplementary material for: Syntheses of novel pyridine-based low-molecular-weight luminogens possessing aggregation-induced emission enhancement (AIEE) properties
Source: Beilstein J Org Chem. 2022 May 24;18:580–7. doi: 10.3762/bjoc.18.60 (PMC9152276; doi:10.3762/bjoc.18.60)
Supplement: File 1 — General information, synthesis of 3a,b, and 4a–e, experimental procedure of fluorescence, theoretical computation method measurements, NMR, UV–vis, and fluorescence spectra. [file Beilstein_J_Org_Chem-18-580-s001.pdf]

BEILSTEIN JOURNAL OF ORGANIC CHEMISTRY

Supporting Information

for

Syntheses of novel pyridine-based low-molecular-weight luminogens possessing aggregation-induced emission enhancement (AIEE) properties

Masayori Hagimori, Tatsusada Yoshida, Yasuhisa Nishimura, Yukiko Ogawa and Keitaro Tanaka

Beilstein J. Org. Chem. **2022,** *18,* 580–587. doi:10.3762/bjoc.18.60

General information, synthesis of 3a,b, and 4a–e, experimental procedure of fluorescence, theoretical computation method measurements, NMR, UV–vis, and fluorescence spectra

License and Terms: This is a supporting information file under the terms of the Creative Commons Attribution License (https://creativecommons.org/licenses/by/4.0). Please note that the reuse, redistribution and reproduction in particular requires that the author(s) and source are credited and that individual graphics may be subject to special legal provisions.
The license is subject to the *Beilstein Journal of Organic Chemistry* terms and conditions: (https://www.beilstein-journals.org/bjoc/terms)

Table of contents

1. General information	S1
2. Synthesis of **3a**,**b**, and **4a**–**e**	S1
3. Experimental procedure of fluorescence measurements	S3
4. Theoretical computation methods	S3
5. NMR spectra (Figures S1–S14)	S4
6. UV–vis absorption spectra (Figures S15–S17)	S11
7. Fluorescence spectra (Figures S18–S20)	S12
8. References	S14

1. General information

The prepared compounds were identified based on NMR spectra recorded on a Gemini 300 NMR (300 MHz), a JEOL JNM-AL400 (400 MHz), and a JEOL JNM ECP500 (500 MHz) instrument, using tetramethylsilane as the internal standard. Mass spectra were recorded on a JEOL DX-303 mass spectrometer. FTIR spectra were recorded on a Shimadzu IRAffinity-1. UV absorption spectra were measured using a Shimadzu UV-2450 spectrophotometer. Fluorescence properties were determined by recording their fluorescence spectra on a Shimadzu RF-5300 pc spectrofluorometer. Melting points were measured using a Yanaco MP-500D melting point apparatus. All chemicals were of reagent grade and were used as received without further purification unless otherwise specified.

2. Synthesis of 3a,b, and 4a–e

2.1. 10-Imino-2-methylpyrido[1,2-*a*]pyrrolo[3,4-*d*]pyrimidine-1,3(2*H*,10*H*)-dione (3a)

A solution of 0.287 g (3.0 mmol) of 1-methyl-4-(methylsulfanyl)-2,5-dioxo-2,5-dihydro-1*H*-pyrrole-3-carbonitrile (**1**) [1,2] and 0.541 g (3.0 mmol) of 2-aminopyridine (**2a**) in 30 mL of ethanol was refluxed for 2 h. After cooling, the precipitate that appeared was collected by filtration to give 0.664 g (2.91 mmol) orange needles, mp 193-194 °C, in 97% yield. ^{1}H NMR (300 MHz, DMSO-d_6) δ: 2.96 (3H, s, NMe), 7.25 (1H, dd, *J* = 2.4, 4.9 Hz, 8-H), 7.54 (1H, d, *J* = 8.2 Hz, 5-H), 7.88 (1H, dd, *J* = 1.1, 1.8, 8.2 Hz, 6-H), 8.41 (1H, ddd, *J* = 1.1, 4,9, 6.0 Hz, 7-H), 11.36 (1H, s, NH). ^{13}C NMR (75 MHz, $CDCl_3$) δ: 59.0, 113.7, 147.8, 150.5, 152.0, 176.0, 177.5, 182.0, 183.9. IR (KBr, cm^{-1}): 3286 (HN=), 1699 (CO), 1664 (CO). Ms: m/z 229 (M^++1, 14), 228 (M^+, 100), 136 (17), 143 (96), 78 (86). *Anal.* Calcd for $C_{11}H_8N_4O_2$=228.0647: C, 57.89; H, 3.53; N, 24.55. Found: C, 57.88; H, 3.32; N, 24.92.

2.2. 10-Imino-2,7-dimethylpyrido[1,2-*a*]pyrrolo[3,4-*d*]pyrimidine-1,3(2*H*,10*H*)-dione (3b)

This compound was prepared in 86% yield from 0.18 g (1.0 mmol) of **1** and 0.11 g (1.0 mmol) of 5-methylpyridin-2-amine (**2b**) in a manner similar to that described for synthesis of **3a**. An analytical sample was recrystallized from methanol to give orange needles, mp 180-181 °C. ^{1}H NMR (300 MHz, DMSO-d_6) δ: 2.31 (3H, s, 7-Me), 2.95 (3H, s, NMe), 7.44 (1H, d, *J* = 6.0 Hz, 8-H), 7.71 (1H, dd, *J* = 2.3, 6.0 Hz, 6-H), 7.99 (1H, d, *J* = 2.3 Hz, 5-H), 11.23 (1H, s, NH). ^{13}C NMR (75 MHz, $CDCl_3$) δ: 51.9, 58.9, 112.1, 148.0, 149.8, 164.9, 173.9, 177.7, 181.4, 182.7. IR (KBr, cm^{-1}): 3277 (HN=), 1701 (CO), 1685 (CO). Ms: m/z 242 (M^+). HRMS (EI): 242.0804 (Calcd. 242.0804 for $C_{12}H_{10}N_4O_2$).

2.3. 4-((5-Bromopyridin-2-yl)amino)-1-methyl-2,5-dioxo-2,5-dihydro-1*H*-pyrrole-3-carbonitrile (4a)

This compound was prepared in 72% yield from 0.18 g (1.0 mmol) of **1** and 0.17 g (1.0 mmol) of 2-amino-5-bromopyridine (**2c**) in a manner similar to that described for synthesis of **3a**. An analytical sample was recrystallized from methanol to give yellow needles, mp 252-253 °C. ^{1}H NMR (400 MHz, DMSO-d_6) δ: 2.96 (3H, s, NMe), 7.51 (1H, d, J = 8.7 Hz, 3-H), 8.12 (1H, d, J = 8.7 Hz, 4-H), 8.48 (1H, s, 6-H), 11.46 (1H, s, NH). ^{13}C NMR (100 MHz, DMSO-d$_6$) δ: 24.3, 79.0, 113.2, 115.9, 117.2, 141.4, 142.8, 147.3, 149.2, 165.3, 169.0. IR (KBr, cm^{-1}): 3221 (HN=), 2222 (CN), 1708 (CO), 1654 (CO). Ms: m/z 306 (M^++1). HRMS (EI): 305.9753 (Calcd. 305.9752 for $C_{11}H_7BrN_4O_2$).

2.4. 4-((5-Fluoropyridin-2-yl)amino)-1-methyl-2,5-dioxo-2,5-dihydro-1*H*-pyrrole-3-carbonitrile (4b)

This compound was prepared in 68% yield from 0.18 g (1.0 mmol) of **1** and 0.11 g (1.0 mmol) of 2-amino-5-fluoropyridine (**2d**) in a manner similar to that described for synthesis of **3a**. An analytical sample was recrystallized from methanol to give yellow needles, mp 220-221 °C. ^{1}H NMR (400 MHz, DMSO-d$_6$) δ: 2.95 (3H, s, NMe), 7.59 (1H, d, J = 5.8 Hz, 3-H), 7.87 (1H, d, J = 5.8 Hz, 4-H), 8.38 (1H, s, 6-H), 11.63 (1H, s, NH). ^{13}C NMR (100 MHz, DMSO-d$_6$) δ: 24.3, 77.7, 113.3, 117.3, 126.4, 134.2, 134.5, 143.3, 146.6, 165.3, 169.2. IR (KBr, cm^{-1}): 3226 (HN=), 2218 (CN), 1718 (CO), 1654 (CO). Ms: m/z 246 (M^+). HRMS (EI): 246.0553 (Calcd. 246.0553 for $C_{11}H_7FN_4O_2$).

2.5. 6-((4-Cyano-1-methyl-2,5-dioxo-2,5-dihydro-1*H*-pyrrol-3-yl)amino)nicotinonitrile (4c)

This compound was prepared in 32% yield from 0.18 g (1.0 mmol) of **1** and 0.12 g (1.0 mmol) of 2-amino-5-cyanopyridine (**2e**) in a manner similar to that described for synthesis of **3a**. An analytical sample was recrystallized from methanol to give yellow needles, mp 208-209 °C. ^{1}H NMR (500 MHz, DMSO-d$_6$) δ: 2.92 (3H, s, NMe), 7.67 (1H, d, J = 11.0 Hz, 3-H), 8.31 (1H, d, J = 11.0 Hz, 4-H), 8.82 (1H, s, 6-H), 11.64 (1H, s, NH). ^{13}C NMR (125 MHz, DMSO-d$_6$) δ: 24.3, 81.6, 104.6, 112.9, 115.0, 116.8, 142.0, 142.1, 150.7, 152.8, 165.3, 168.6. IR (KBr, cm^{-1}): 3213 (HN=), 2225 (CN), 2218 (CN), 1718 (CO), 1685 (CO). Ms: m/z 253 (M^+). HRMS (EI): 253.0599 (Calcd. 253.0600 for $C_{12}H_7N_5O_2$).

2.6. Methyl 6-((4-cyano-1-methyl-2,5-dioxo-2,5-dihydro-1*H*-pyrrol-3-yl)amino)nicotinate (4d)

This compound was prepared in 43% yield from 0.18 g (1.0 mmol) of **1** and 0.15 g (1.0 mmol) of methyl-6-aminonicotinate (**2f**) in a manner similar to that described for synthesis of **3a**. An analytical sample was recrystallized from methanol to give orange needles, mp 245-246 °C. ^{1}H NMR (400 MHz, DMSO-d$_6$) δ: 2.95 (3H, s, NMe), 3.87 (3H, s, OMe), 7.62 (1H, d, J = 8.8 Hz, 3-H), 8.31(1H, d, J = 7.3 Hz, 4-H), 8.84 (1H, s, 6-H), 11.64 (1H, s, NH). ^{13}C NMR (100 MHz, DMSO-d$_6$) δ: 24.4, 52.4, 112.8, 114.7, 114.8, 121.8, 139.5, 147.9, 153.4, 164.6, 165.5, 169.0. IR (KBr, cm^{-1}): 3221 (HN=), 2225 (CN), 1715 (CO), 1685 (CO). Ms: m/z 286 (M^+). HRMS (EI): 286.0701 (Calcd. 286.0702 for $C_{13}H_{10}N_4O_4$).

2.7. Methyl 1-methyl-2,5-dioxo-4-((5-(trifluoromethyl)pyridin-2-yl)amino)-2,5-dihydro-1*H*-pyrrole-3-carbonitrile (4e)

This compound was prepared in 46% yield from 0.18 g (1.0 mmol) of **1** and 0.16 g (1.0 mmol) of 2-amino-5-(trifluoromethyl)-pyridine (**2g**) in a manner similar to that described for synthesis of **3a**. An analytical sample was recrystallized from methanol to give yellow needles, mp 216-217 °C. ^{1}H NMR (400 MHz, DMSO-d_6) δ: 2.95 (3H, s, NMe), 7.70 (1H, d, *J* = 8.3 Hz, 3-H), 8.26 (1H, d, *J* = 6.3 Hz, 4-H), 8.67 (1H, s, 6-H), 11.65 (1H, s, NH). ^{13}C NMR (100 MHz, DMSO-d_6) δ: 24.4, 81.0, 113.1, 115.2, 121.1, 124.8, 131.5, 142.4, 143.8, 153.4, 165.4, 168.9. IR (KBr, cm^{-1}): 3170 (HN=), 2222 (CN), 1718 (CO), 1647 (CO). Ms: m/z 296 (M^+). HRMS (EI): 296.00521 (Calcd. 296.0521 for $C_{12}H_7F_3N_4O_2$).

3. Experimental procedure of fuorescence measurements

Each compound was dissolved in dimethyl sulfoxide (DMSO) to prepare stock solutions (10^{-2} mol/L). The concentration of each sample was adjusted using a molar absorption coefficient of 0.05. The excitation wavelength was determined by scanning the emission wavelength. In a similar manner, the emission wavelength was obtained by scanning the excitation wavelength. Fluorescence quantum yields were determined using the Absolute PL Quantum Yield Measurement System (C9920-01) of Hamamastu Photonics (Shizuoka, Japan).

4. Theoretical computation methods

Density functional theory (DFT)-based calculations were performed on compounds **3a** and **4e** using the Gaussian 16 program [3]. To geometrically optimize the monomer structure of each compound in the ground state, we used Becke's three-parameter hybrid exchange functional with the Lee-Yang-Parr gradient-corrected correlation (B3LYP) functional suitable for small organic molecules and the 6-31G(d,p) basis set. To evaluate the excitation energies, single-point energy calculations using time-dependent (TD) DFT were performed on ground-state optimized structures at the TD-B3LYP/6-311+G(d,p) level of theory. Both optimization and TDDFT calculations were performed in different solvent environments (dichloromethane, ethanol, and water) using the polarizable continuum model (PCM) based on the linear response approach.

5. NMR spectra (Figures S1–S14)

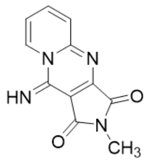

3a

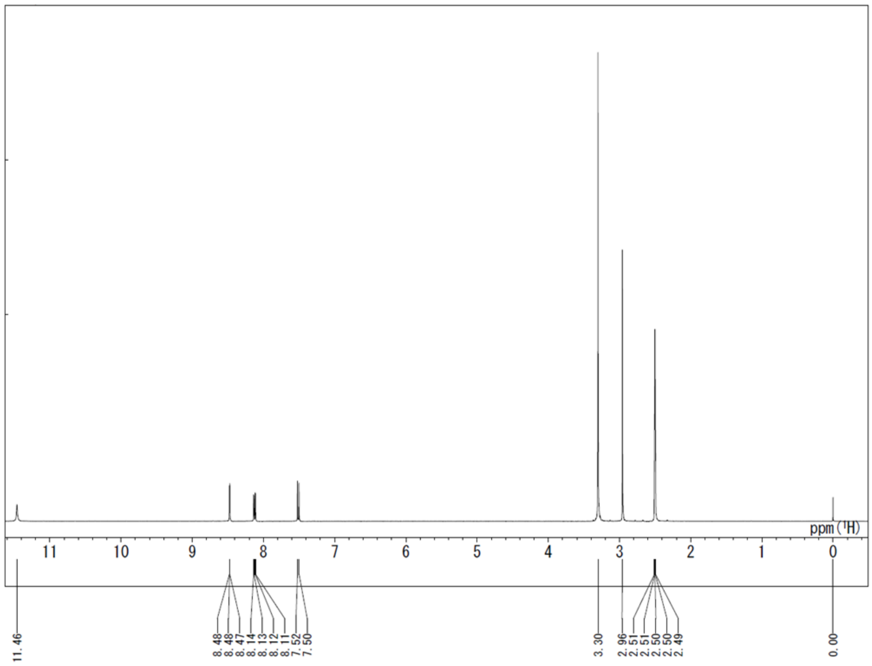

Figure S1. ^{1}H NMR spectrum of **3a**

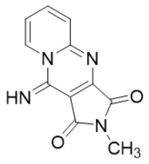

3a

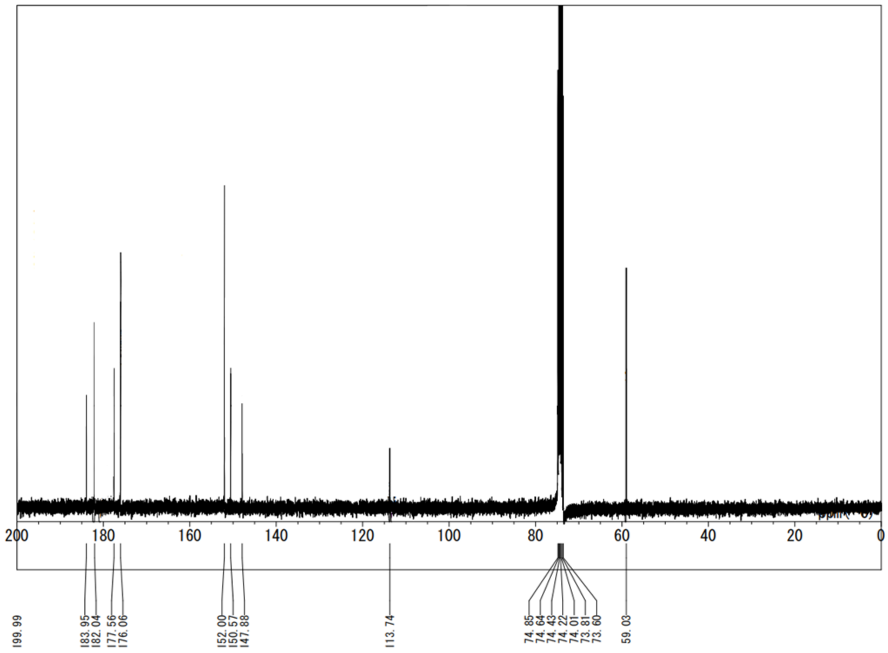

Figure S2. ^{13}C NMR spectrum of **3**a

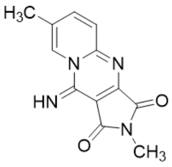

3b

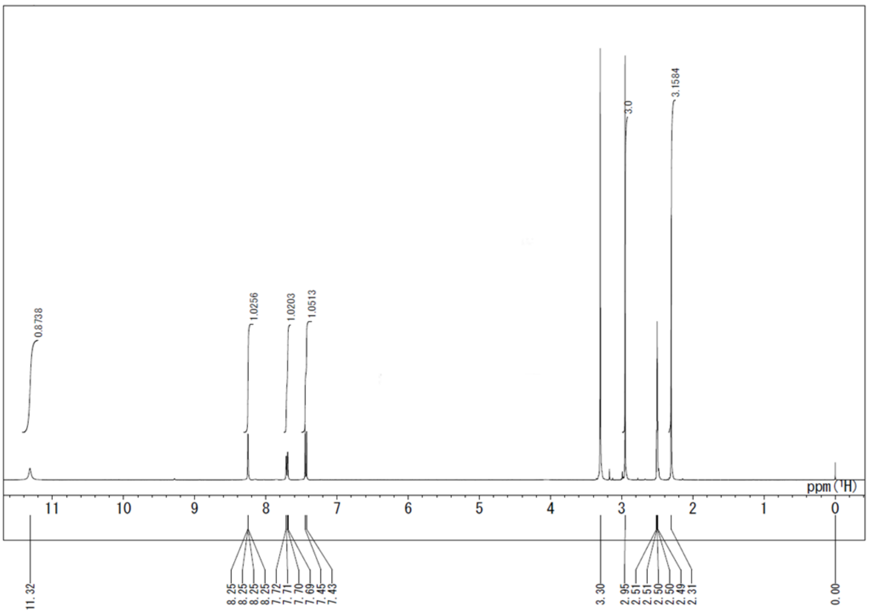

Figure S3. ^{1}H NMR spectrum of **3b**

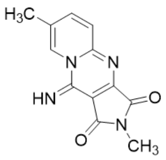

3b

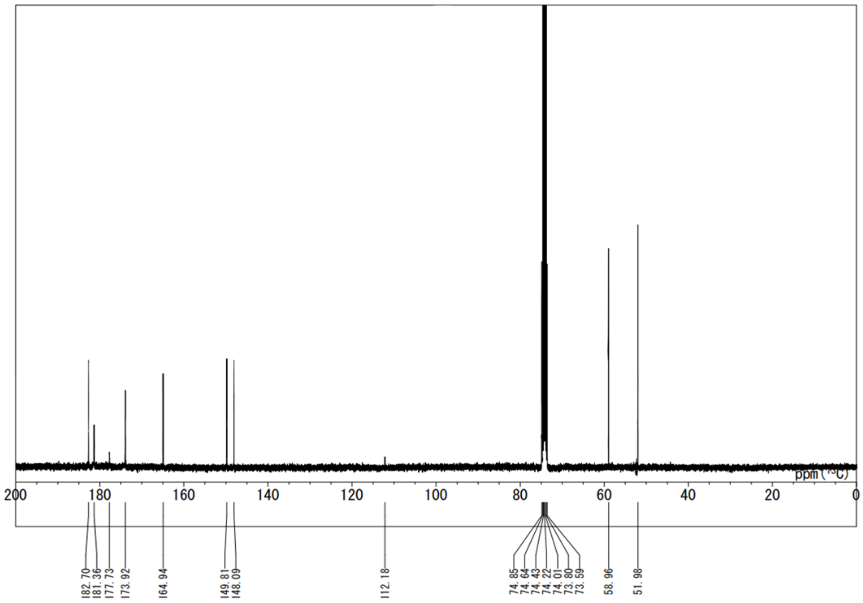

Figure S4. ^{13}C NMR spectrum of **3b**

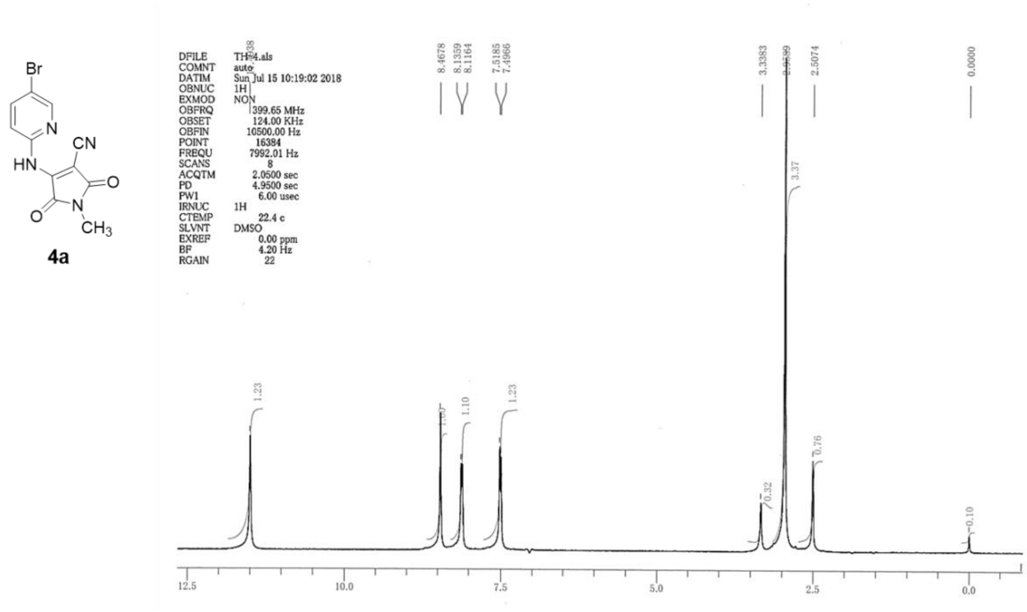

Figure S5. ^{1}H NMR spectrum of **4a**

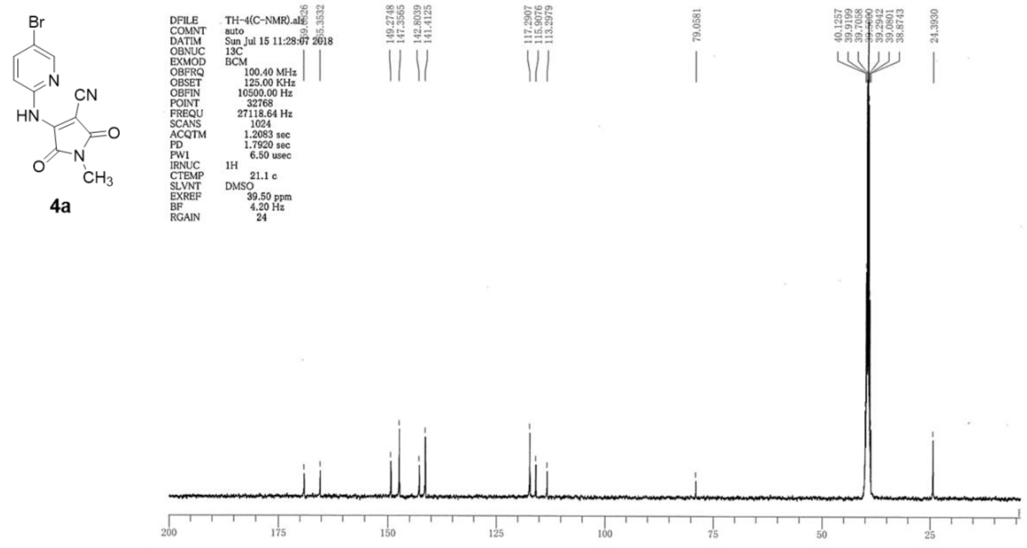

Figure S6. ^{13}C NMR spectrum of **4a**

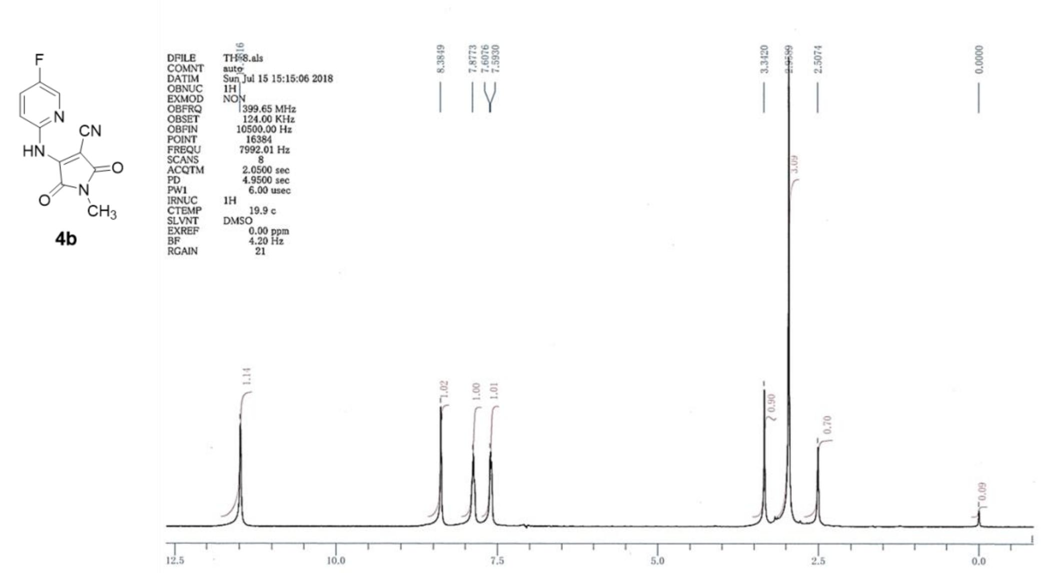

Figure S7. ^{1}H NMR spectrum of **4b**

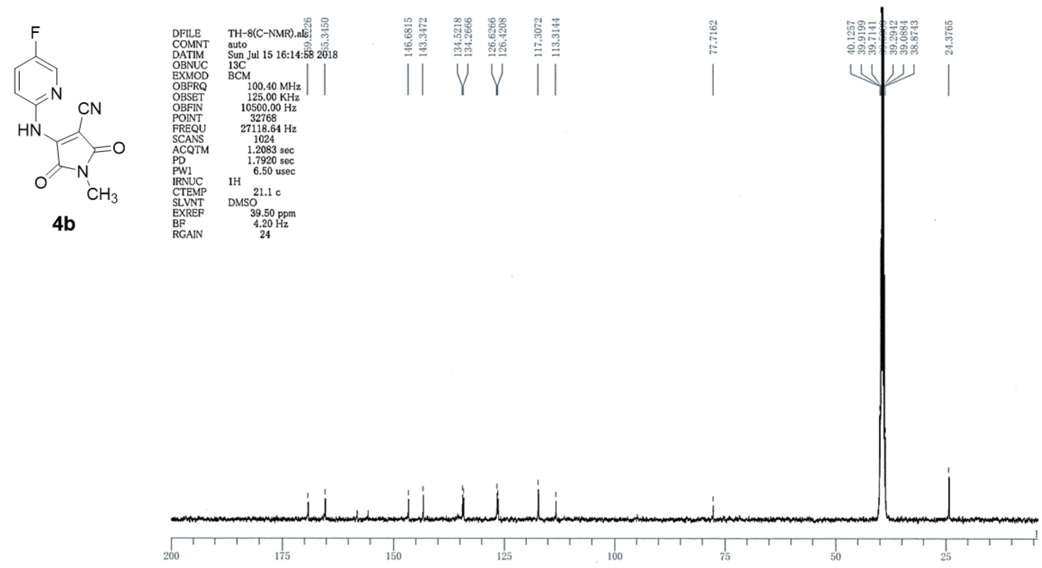

Figure S8. ^{13}C NMR spectrum of **4b**

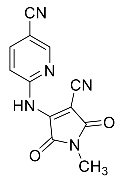

4c

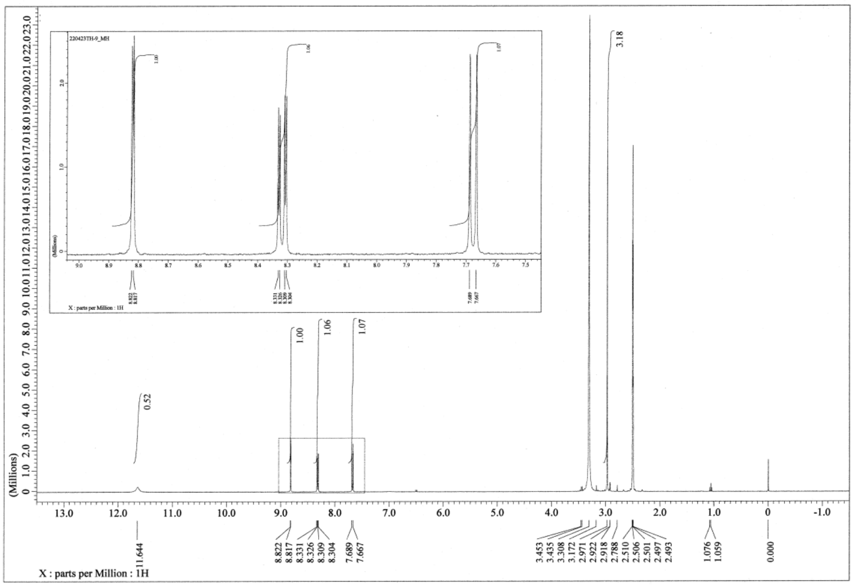

Figure S9. ^{1}H NMR spectrum of **4c**

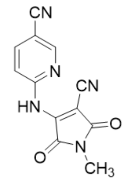

4c

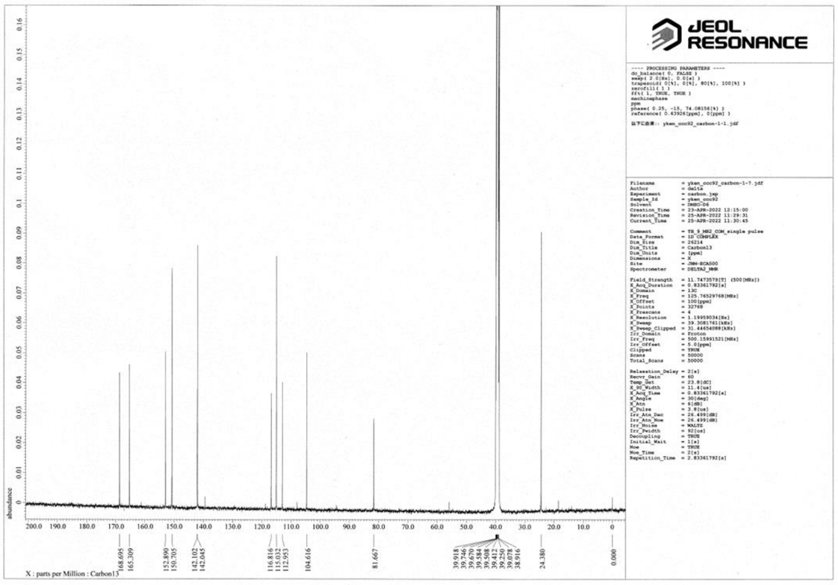

Figure S10. ^{13}C NMR spectrum of **4c**

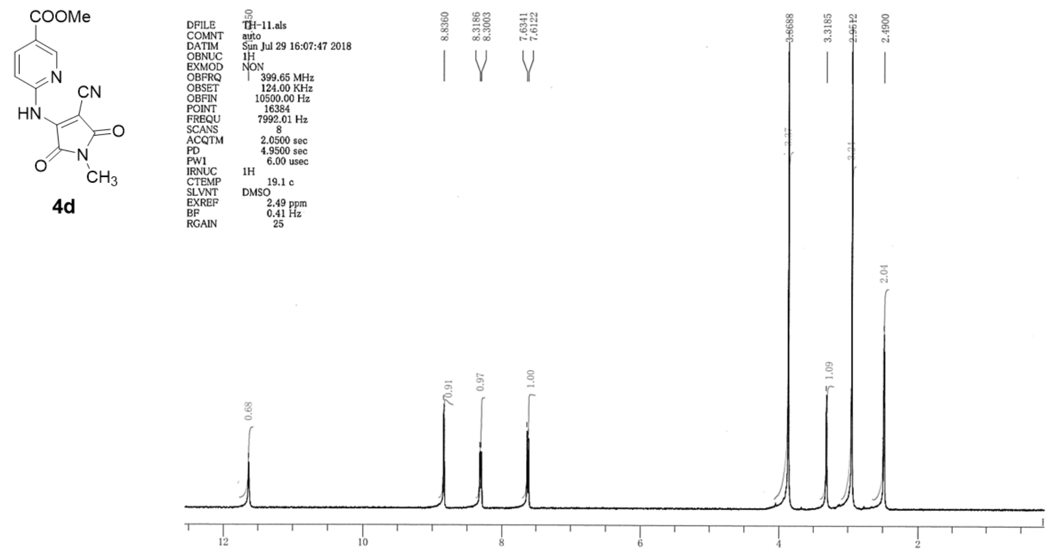

Figure S11. ^{1}H NMR spectrum of **4d**

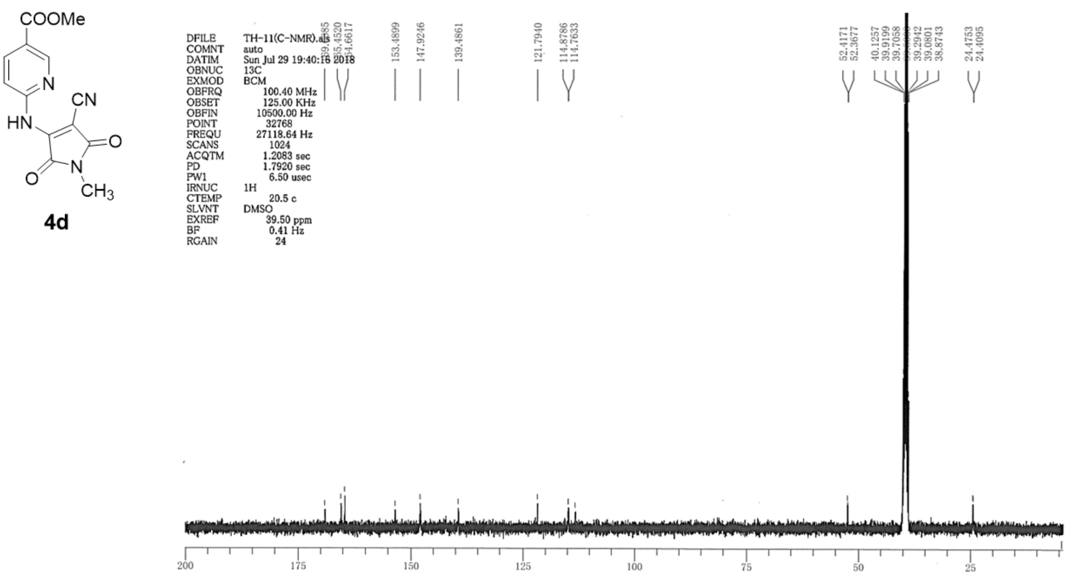

Figure S12. ^{13}C NMR spectrum of **4d**

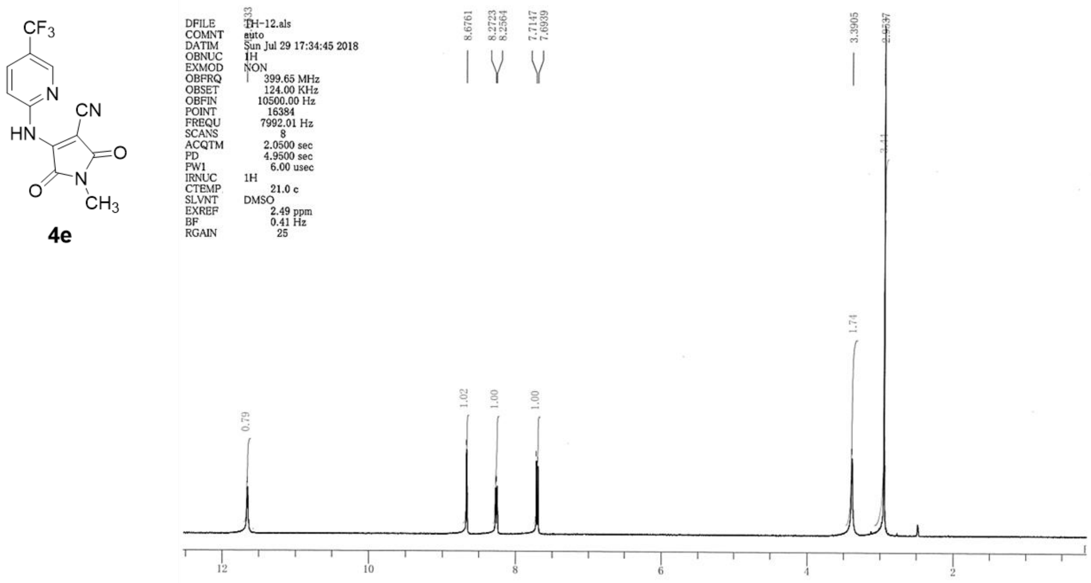

Figure S13. ^{1}H NMR spectrum of **4e**

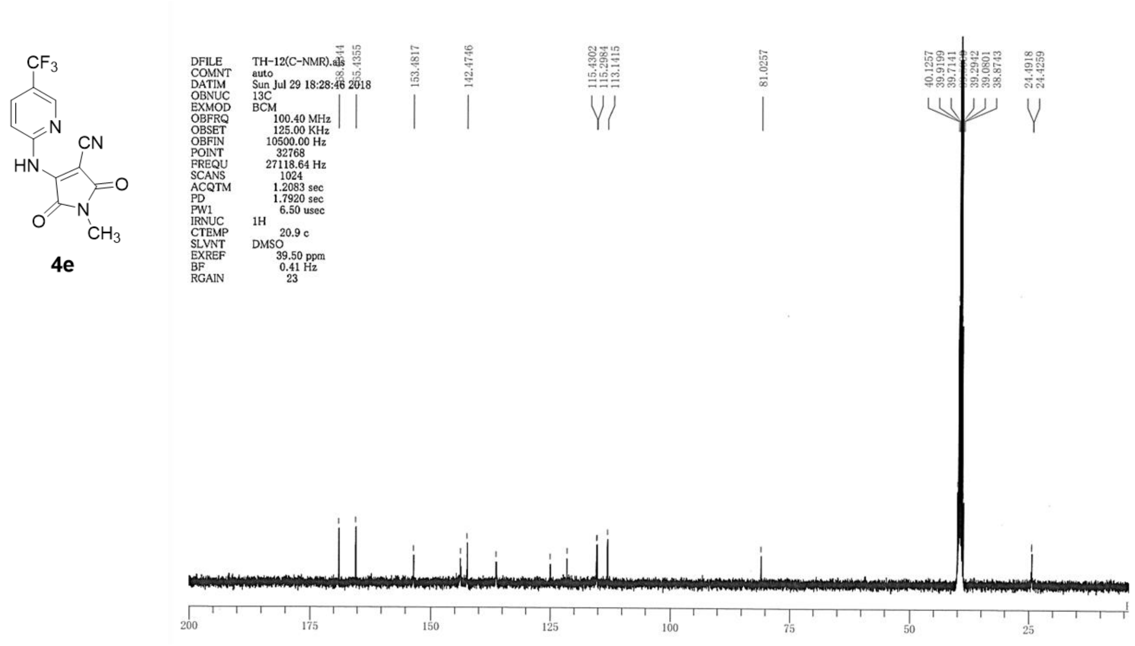

Figure S14. ^{13}C NMR spectrum of **4e**

6. UV–vis absorption spectra (Figures S15–S17)

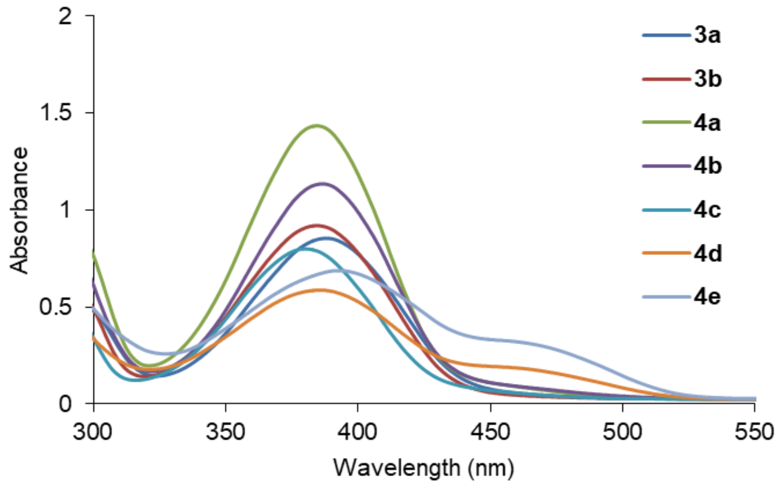

Figure S15. UV–vis absorption spectra of **3a**, **3b**, and **4a**–**e** (10 μM) in EtOH.

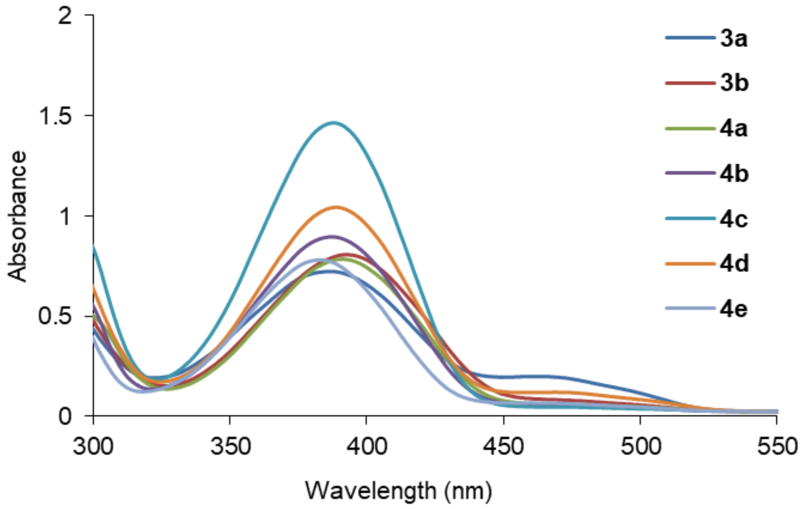

Figure S16. UV–vis absorption spectra of **3a**, **3b**, and **4a**–**e** (10 μM) in DCM.

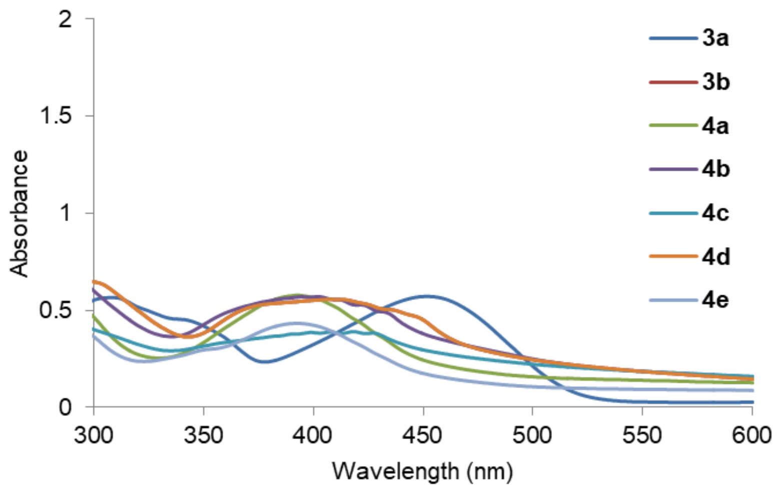

Figure S17. UV/Vis absorption spectra of **3a**, **3b** and **4a–e** (10 μM) in H_2O.

7. Fluorescence spectra (Figures S18-S20)

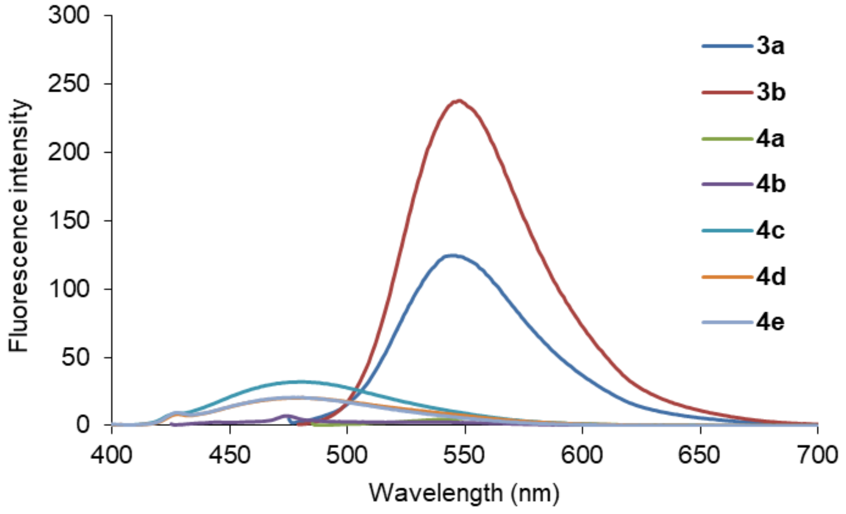

Figure S18. Fluorescence spectra of **3a**, **3b**, and **4a–e** (10 μM) in EtOH.

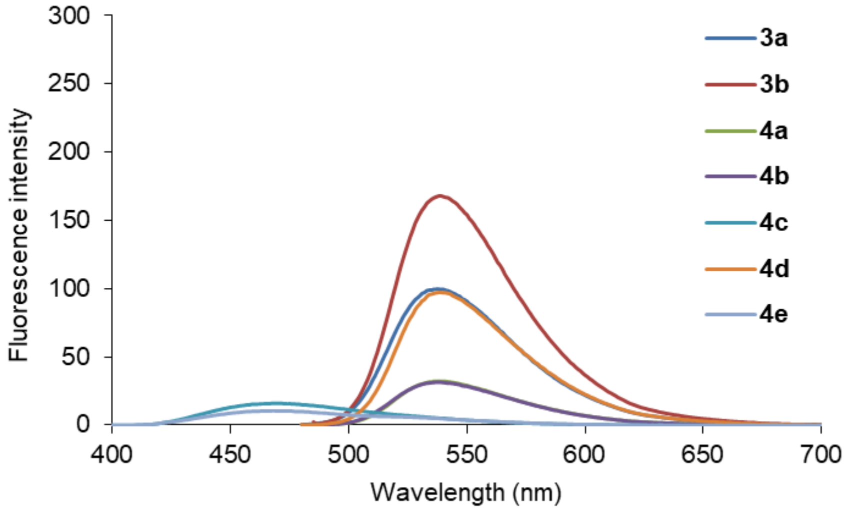

Figure S19. Fluorescence spectra of **3a**, **3b**, and **4a–e** (10 μM) in DCM.

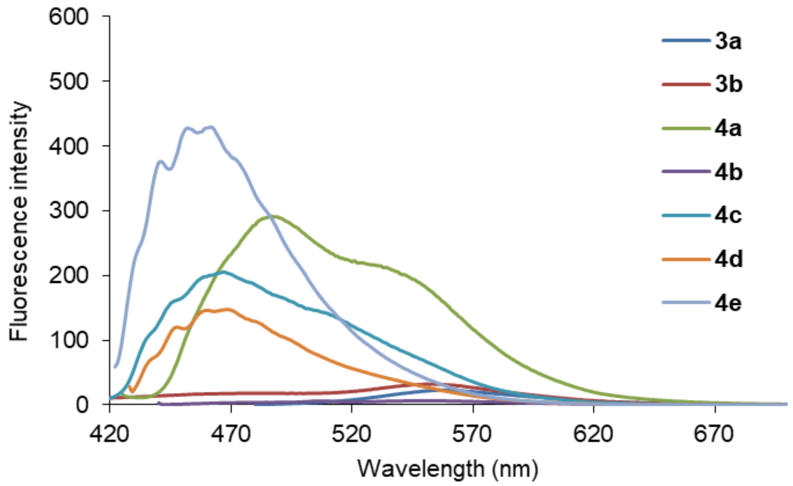

Figure S20. Fluorescence spectra of **3a**, **3b**, and **4a–e** (10 μM) in H_2O.

8. References

1. Shigemitsu, Y.; Komiya, K.; Mizuyama, N.; Hagimori, M.; Tominaga, Y. *Res. Lett. Org. Chem.* **2009**, 2009, 1–5.

2. Hagimori, M.; Mizuyama, N.; Yokota, K.; Morinaga, O.; Yamaguchi, Y.; Saji, H.; Tominaga, Y. *Heterocycles* **2011**, 83, 1983–1988.

3. Frisch, M. J.; Trucks, G. W.; Schlegel, H. B.; Scuseria, G. E.; Robb, M. A.; Cheeseman, J. R.; Scalmani, G.; Barone, V.; Petersson, G. A.; Nakatsuji, H.; Li, X.; Caricato, M.; Marenich, A. V.; Bloino, J.; Janesko, B. G.; Gomperts, R.; Mennucci, B.; Hratchian, H. P.; Ortiz, J. V.; Izmaylov, A. F.; Sonnenberg, J. L.; Williams-Young, D.; Ding, F.; Lipparini, F.; Egidi, F.; Goings, J.; Peng, B.; Petrone, A.; Henderson, T.; Ranasinghe, D.; Zakrzewski, V. G.; JGao, J.; Rega, N.; Zheng, G.; Liang, W.; Hada, M.; Ehara, M.; Toyota, K.; Fukuda, R.; Hasegawa, J.; Ishida, M.; Nakajima, T.; Honda, Y.; Kitao, O.; Nakai, H.; Vreven, T.; Throssell, K.; Montgomery Jr., J. A.; Peralta, J. E.; Ogliaro, F.; Bearpark, M. J.; Heyd, J. J.; Brothers, E. N.; Kudin, K. N.; Staroverov, V. N.; Keith, T. A.; Kobayashi, R.; Normand, J.; Raghavachari, K.; Rendell, A. P.; Burant, J. C.; Iyengar, S. S.; Tomasi, J.; Cossi, M.; Millam, J. M.; Klene, M.; Adamo, C.; Cammi, R.; Ochterski, J. W.; Martin, R. L.; Morokuma, K.; Farkas, O.; Foresman, J. B.; Fox, D. J. Gaussian16, Revision A.03. Gaussian, Inc., Wallingford, CT, **2016**.
